# Triple-negative breast cancer: current treatment strategies and factors of negative prognosis

**DOI:** 10.25122/jml-2021-0108

**Published:** 2022-02

**Authors:** Anna Baranova, Mykola Krasnoselskyi, Volodymyr Starikov, Sergii Kartashov, Igor Zhulkevych, Vadym Vlasenko, Kateryna Oleshko, Olga Bilodid, Marina Sadchikova, Yurii Vinnyk

**Affiliations:** 1. Department of Radiology and Oncology, Grigoriev Institute for Medical Radiology NAMS of Ukraine, Kharkiv, Ukraine; 2. Department of Oncology, Kharkiv National Medical University, Kharkiv, Ukraine; 3. Department of Oncology, Radiology and Radiation Medicine V. N. Karazin Kharkiv National University, Kharkiv, Ukraine; 4. Department of Obstetrics, Gynecology and Oncogynecology, Kharkiv Medical Academy of Postgraduate Education, Kharkiv, Ukraine; 5. Department of Oncology, Radiology Diagnostics and Therapy and Radiation Medicine, I. Horbachevsky Ternopil National Medical University, Ternopil, Ukraine; 6. Department of Cancer Surgery, Medical Center Molecule, Kharkiv, Ukraine; 7. Department of Cancer Surgery, Radiation Therapy and Palliative Care, Kharkiv Medical Academy of Postgraduate Education, Kharkiv, Ukraine

**Keywords:** breast cancer, triple-negative type, surgical treatment, radical mastectomy, organ-preserving operations, late result, ART – adjuvant radiation therapy, AR – androgen receptors, ACT – adjuvant chemotherapy, IHC – immunohistochemical, BMI – body mass index, LN – lymph node, NACT – neoadjuvant chemotherapy, ER – estrogen receptors, RM – radical mastectomy, BC – breast cancer, TNBC – triple-negative breast cancer, CT– chemotherapy

## Abstract

Breast cancer is the most common cancer in women and the most common cause of death in working-age women. According to the results of immunohistochemical studies, 10–20% of cases revealed a triple-negative type of breast cancer. This subtype is characterized by significant proliferative activity and growth rate, aggressive clinical course, and early metastasis. This leads to a suspicious prognosis and, accordingly, encourages an increase of surgical treatment radicalism and aggressive systemic treatment. This review briefly analyzes existing treatment strategies for triple-negative breast cancer with a focus on surgical treatment. Surgical treatment is an integral part of complex therapy. Currently, the attention of researchers is focused not only on the radicalism of the operation, ensuring long-term survival, but also on achieving a good cosmetic result that determines the quality of life of patients. In this aspect, organ-preserving and prosthetic methods of operations are promising, the feasibility and effectiveness of which are being discussed. The relevance of choosing the optimal method of operation is evidenced by the lack of generally accepted approaches based on informative markers for the prognosis of the course of the disease. Therefore, the choice of the optimal method of surgical treatment taking into account the individual characteristics of the patient and the tumor, indications for chemotherapy, and radiation therapy remains an unresolved issue and requires further research.

## Introduction

### Epidemiology and clinical features of triple-negative breast cancer

In recent years, breast cancer (BC) has confidently taken a leading position in the structure of morbidity and mortality from cancer in most countries of Eastern and Western Europe, Asia, and America. According to a study by the global project GLOBOCAN, it is the most common cancer in women, accounting for 25.1% of all cancers [1–3]. BC is a very heterogeneous disease with a different course, prognosis, sensitivity to therapy, and other characteristics, due to the variety of genetic aberrations against this disease [4]. Most often, an immunohistochemical (IHC) study is used to determine the subtype of the disease, which allows determining the level of expression of receptors on the surface of tumor cells – estrogen receptors (ER), progesterone receptors (PR), and oncoprotein HER-2/neu (human epidermal growth factor receptor 2 – the second epidermal growth factor receptor). One of the most difficult to treat is triple-negative breast cancer (TNBC), in which ER, PR, HER-2/neu are absent or detected in very low concentrations [4, 5]. This subtype is detected in 10–20% of cases and is characterized by significant proliferative activity and growth rate, aggressive clinical course, early metastasis, and negative prognosis [6–8]. It should be noted that TNBC is also not homogeneous. Fundamental genetic research has established a significant heterogeneity of TNBC. Thus, according to gene expression analysis in 587 cases of TNBC Lehmann *et al.* identified 6 subtypes: two basal-like, immunomodulatory, mesenchymal, mesenchymal stem, and one with androgen receptors [9].

According to Fayaz *et al.*, TNBC represents 12% of the structure of BC. Stage 1 disease is set in 15%, stage 2 – in 43%, stage 3 – in 35% and stage 4–7% of patients. Lesions of subclavian lymph nodes (LN) were found in 82% of patients [7]. According to Kümmel *et al.*, among 3,054 women with breast cancer, the triple-negative subtype was detected in 11% of cases [10]. Sharma *et al.* found TNBC in 15% of cases and noted that it is associated with a poor prognosis. Almost 75% of these cancers are basal carcinomas, and by histotype belong to low-grade ductal carcinomas [8]. The frequency of locoregional recurrences of TNBC is similar to the HER2+ subtype and almost 50% higher than in the luminal subtype [11]. A comparative analysis of the clinical course of breast cancer in 321 women with TNBC and 1,212 women with other breast cancer subtypes found that in TNBC, there was a higher stage of T, N, a lower degree of histological differentiation [12].

According to the results of the analysis of the German Cancer Registry, it was found that in women diagnosed with breast cancer during 10 years of observation, locoregional recurrences were detected in 8%, and distant metastases in 11% of women. The frequency of recurrence and metastasis in TNBC was 23%, which allowed identifying this subtype as an important risk factor [13]. Other researchers also identify triple-negative status as a risk factor for locoregional recurrence [14]. In a systematic review, there were significant differences in the frequency of locoregional recurrences depending on the tumor subtype – the smallest in luminal A (1.7%), the largest in TNBC (7.4%), but this only applied to patients taking Trastuzumab [15]. James M. *et al.* reported that the 5-year overall survival in TNBC was 72%, with a life expectancy of 3.55 years. The cause of recurrence in 74% of patients were distant metastases (55.9% – in the lungs), local recurrences – in 4.5% of patients [16]. In an earlier study, the lowest 5-year survival was observed in TNBC, amounting to 62.1%, the frequency of distant recurrences reached 35.2%, locoregional – 4.2% [17]. According to the analysis of 111 cases of TNBC, Bayoumi Y. *et al.* found that 68.5% of patients were diagnosed with stage III disease, and 73% – lymph node (LN) lesions. The 5-year survival for the whole group of patients was (54±8)% [18], and according to Fayaz S. *et al.* (2019) the 10-year survival of patients with TNBC was: total – 66% (with a clear dependence on the stage of the disease: stage 1 – 92%, stage 2 – 80%, stage 3 – 49%, stage 4 – 0), recurrence-free – 59%, without distant metastases – 72%, without local recurrence – 77% [7].

In a study that analyzed the frequency of local and locoregional metastases in 335 patients who underwent organ-sparing surgery and neoadjuvant therapy for stage 2–3 breast cancer, TNBC was detected in 61 patients (18.2%). At TNBC, the 5-year survival rates without locoregional and local recurrences were the lowest compared to other subtypes (79.6% and 84.6%, respectively). Locoregional recurrences were detected in 21.3% (most often in subclavian and intramammary lymph nodes), local – in 14.8%, distant metastases – in 29.5% of patients with this subtype of breast cancer. According to the multivariate analysis, the triple-negative subtype had the highest risk of local recurrence [19].

The aim of this review was to compare existing methods of surgical and systemic treatment of triple-negative breast cancer and the factors that determine their effectiveness. We searched for articles in the Pubmed database from 2010 to 2020 by combining keywords: breast cancer, triple-negative type, surgical treatment, radical mastectomy, organ-preserving operations, late result. Articles corresponding to the aim of the review were selected.

## Results

### Basic treatment methods for triple-negative breast cancer

General principles and methods of treatment of TNBC are similar to treatment of other subtypes of cancer of this localization. The main components of complex or combined treatment are surgical removal of the tumor and areas of regional metastasis, topical treatment with radiation therapy and chemotherapy (CT) in neoadjuvant (NACT) and/or adjuvant regimens. The range of surgeries is very wide, from a radical mastectomy (RM) to organ-saving operations, biopsy of sentinel LN with a reasonable refusal of lymph dissection [6]. However, due to the peculiarities of the clinical course and prognosis of TNBC, treatment strategies differ in more active oncosurgical tactics and systemic treatment. Until recently, radical mastectomy (RM) was the main surgical intervention in TNBC.

However, after RM, in addition to the fairly frequent development of lymphedema and other postoperative complications, there is a decrease in self-esteem, loss of femininity, attractiveness, and sexuality with the development of psycho-emotional disorders [20]. Therefore, in recent years, organ-preservation techniques of surgical interventions have been actively developed, during which it is also necessary to adhere to the principles of oncological radicalism to prevent local and regional recurrences of the disease. This requires, in many cases, the removal of a large area of the breast, which leads to significant deformations in the body and unsatisfactory aesthetic results, being observed in almost 2/3 of cases [21].

#### Organ-sparing surgery of TNBC

In addition to oncological radicalism, the cosmetic result of the surgery is of great importance, which is especially relevant due to the younger age of patients with TNBC, given the impact on the quality of life of patients. In this aspect, the development of oncological surgery of the breast took place in two main directions: reduction of indications for BC in favor of organ-saving operations and development of reconstructive and restorative operations.

Umberto Veronesi is considered to be a pioneer of organ-sparing surgery, who in the ‘80s of the last century identified the quality of life as the main principle of treatment of patients with breast cancer and proposed quadrantectomy and histological examination of the sentinel (closest to the tumor) lymph node, the results of which determined the need for more radical surgeries. According to him, surgical treatment should be more conservative to ensure a better quality of life while achieving good medical results [22].

In order to maximize the preservation of own tissues, skin-preserving mastectomy and mastectomy with preservation of the nipple-areolar complex (NAC) are used. Preservation of these structures facilitates the further reconstruction of soft tissues, the frequency of recurrences with these methods does not differ from the results of RM [23–25]. To reduce the trauma of the surgery and achieve a better cosmetic result in recent years, endoscopic or endoscopically-assisted subcutaneous mastectomy has been proposed, which allows avoiding large incisions and preserving the skin, NAC, and submammary fold. These operations are considered appropriate in the early stages of breast cancer, with the central location of the tumor, the small size of the breast in the absence of its ptosis [26–28]. Lai HW *et al.* report the results of 315 endoscopic skin-preserving mastectomies in patients with early breast cancer. Postoperative complications were observed in 15.2% of cases, the frequency of local recurrence – in 1% of cases. Most patients had a good cosmetic result [27]. The results of endoscopic mastectomy with simultaneous implant reconstruction did not differ from similar open-access operations but provided a better cosmetic result in early breast cancer (median follow-up 74 months) [29].

#### Autoplastic breast surgery

Both after organ-preserving surgeries and after RM, restoration of the size, shape, and natural contours of the breast is possible only with the help of reconstructive plastic surgery. Restoration of natural breast contours is carried out using own tissues (autotransplantation, flap reconstruction) or artificial materials – endoprostheses (allotransplantation). Free, vascularized, and revascularized flaps are used as autografts in a plastic mastectomy. The most commonly used myocutaneous flaps on the nourishing vascular pedicle: from the widest muscle of the back (m. Latissimus dorsi – LDM-flap), from the rectus abdominis (m. transverse rectus abdominis – TRAM-flap), less often deep perforated and superficial lower epigastric flaps etc. For reconstruction, the skin flap is excised together with the underlying muscle with the preserved vascular-nerve bundle, which is carried to the area of soft tissue defect after mastectomy [30–32]. In addition, in order to replace the tissue defect after mastectomy, autologous fat is used [33–35].

Autotransplantation methods are technically complex, require existing experience in plastic surgery, microsurgery, careful calculations of the size of transplanted flaps, more traumatic due to additional trauma to the donor site. Therefore, alloplastic implants are much more often used for soft tissue reconstruction after mastectomy. The share of this technology reaches 80%. These methods are technically less complex, and their results are not inferior to autotransplantation [36].

The beginning of modern breast implantology can be considered 1962 when a two-component implant was proposed in the form of an elastomeric silicone shell filled with liquid silicone. Later in 1965, it was proposed to fill the shell with saline [37]. In 1971 Snyderman R. K. and Guthrie R. H. reported the successful results of a one-step reconstruction with a silicone implant located under the skin envelope remaining after mastectomy. To achieve symmetry, a reduction in the contralateral breast was proposed [38].

#### Implants breast reconstruction

There are three main types of breast reconstruction with the help of implants: one-stage implantation of an endoprosthesis in a prepared skin pocket after skin-preserving mastectomy; two-stage implantation using the method of expander dermatosis – preparation of the skin pocket with an expander in the first stage, followed by implantation of an endoprosthesis in the second stage (for 4–6 months); implantation of a permanent expander, which is not removed, but is also an implant consisting of two shells – one filled with silicone, the other – saline, increasing the volume of which provides a gradual stretching of the skin. There are no generally accepted “best” methods of endoprosthesis implantation. There are supporters of one-stage or two-stage reconstruction, subpectoral or prepectoral placement of implants, the use of additional materials to improve the fixation of the endoprosthesis, and other surgery features. The choice of some methods depends on the patient’s characteristics, the size and location of the tumor, which depends on the amount of resection of the breast or mastectomy, the available experience of the surgeon, and other factors.

Primary reconstruction has many supporters because it shortens the recovery time with good immediate and long-term results [39]. However, Nahabedian MY and Jacobson SR noted that most patients undergo two-stage breast reconstruction after mastectomy and believe this method is technically simpler and less traumatic. In women who require postoperative radiation therapy, these authors considered the prepectoral location of the implant to be optimal [40]. Casella D. *et al.* report good results of prepectoral reconstruction of the breast after mastectomy using subcutaneous expanders [41].

Additional materials are used to cover the endoprosthesis and form a submammary fold. The most available material is synthetic mesh, but their use leads to a large number of postoperative complications, in particular, lymphorrhea and capsular contracture. Acellular dermal matrix (ADM), which is offered by several pharmaceutical companies (FlexHD, DermaMatrix, AlloDerm etc), is often used, but it has a high cost, which reaches $3,400 per standard flap. A good alternative is the use of the inferior de-epithelialized flap (IDF) of the breast, which is autologous and is created during surgery [42–44]. The cosmetic outcome and frequency of complications requiring repeated surgery with ADM and IDF did not differ significantly [42], as evidenced by a prospective study by Sorkin M. *et al.* [44]. Nahabedian MY and Jacobson SR believe it is advisable to use an acellular dermal matrix to ensure tissue support and the long-term stability of the implant [40]. Casella D. *et al.* reported good results in the use of synthetic mesh during the reconstruction of the breast [41].

Thus, the arsenal of surgical treatment methods of BC is quite wide. The general trend of recent years is a combination of plastic surgery and oncology – an increase in the proportion of skin-preserving mastectomy with plastic by own tissues or endoprostheses and the rejection of extensive lymph node dissections in favor of sentinel lymph node biopsy. It should be noted that the choice of method of surgical intervention depends on the decisions of the surgeon and patient. According to Rippy *et al.*, 27% of patients refused to perform organ-preserving surgery in favor of mastectomy, but the patients’ choice depends on their understanding of this aspect of treatment [45].

#### Long-term result of TNBC surgery

The choice of surgery remains one of the most difficult problems in the treatment of TNBC [46]. The general trend in BC, including TNBC, is the desire not only for oncological radicalism but also to ensure a good cosmetic result, which is possible with organ-preserving reconstructive plastic surgery. But the clinical and pathological features of TNBC limit the indications for these operations. Due to the doubtful prognosis, in the case of TNBC, mastectomy is performed more often than organ-preserving surgery – 67% and 33%, respectively [7]. According to Bayoumi Y. *et al.* this ratio was 60% and 40% in favor of RM [18]. According to Adkins FC *et al.*, among 1325 patients with TNBC, organ-preserving surgery was performed in 49% of cases, and 51% – mastectomy [47]. However, the choice of surgical treatment depends not so much on the molecular biological subtype but other features of the tumor. In particular, women with TNBC who underwent mastectomy had larger tumor sizes, lymphovascular invasion, and LN lesions [47]. In another study, women who underwent mastectomy were younger, had signs of lymphovascular invasion, and had larger tumors [48]. In a study by Abdulkarim BS *et al.*, RM was associated with lymphovascular invasion and LN lesion [49]. Rezai *et al.* believe that RM is appropriate for ductal tumor, G3 differentiation degree, HER2+, and TNBC subtypes [50]. Mastectomy was performed in 43.4% of women in a large cohort of women with BC (87.504 cases). Age, stage, marital status and race were correlated with the implementation of mastectomy. TNBC was associated with the implementation of contralateral prophylactic mastectomy. Over time, there was a decrease in the frequency of mastectomy, but its frequency increased in BC stage 3 [51]. On the contrary, according to Zumsteg *et al.* (2013), among 646 patients with T1-2N0, 448 (69.3%) underwent resection, and 198 (30.7%) underwent RM [48].

Therefore, in many cases, it is difficult to determine which factors (features of the tumor or surgery) were more influential on treatment results. Most authors do not consider the surgery volume as a prognostic factor [47]. According to Fayaz *et al.*, survival was affected only by stage and lymphovascular infiltration. The volume of surgery did not affect survival [7]. James M. *et al.* consider lymphovascular invasion, nodal status, and tumor size significant prognostic factors [16]. In another study, the risk factors for reoperation in ductal breast cancer were tumor size greater than 20 mm, in invasive breast cancer – age over 40 years. Multifocal tumors, lymphovascular lesions, and HER2+/-status also increased the risk of reoperation [52]. Analysis of the risk of recurrence of TNBC in 390 women found an increase in the frequency of locoregional recurrence in women less than 50 years, in the presence of lymphovascular invasion, stage 3 disease, and lesions of 3 lymph nodes. The five-year frequency of locoregional recurrence in the presence of one risk factor was 4.2%, two – 25.2%, three or four risk factors – 81% [53].

A study based on the analysis of 1035 cases of segmental resections with primary mammoplasty in breast cancer (5 variants of primary mammoplasty) found that locoregional recurrences did not depend on surgical technique and volume of resection, T and N status, and type of tumor (ductal or lobular). Local recurrences were associated with ductal tumor, G3 differentiation, HER2+ and TNBC subtypes [50]. The best results were obtained in patients with luminal A subtype of BC. The authors note that even with a close location of the tumor to the nipple-areola (less than 20 mm) under the condition of optimal antitumor treatment, the results were excellent [54].

However, in another study, according to the analysis of 768 cases of TNBC with an average follow-up of 7.2 years, a better result of resection interventions was obtained compared to RM [49]. But this can be explained by the more severe baseline status of the tumor, which was the basis for more radical surgery.

In addition to the amount of breast tissue removal, the feasibility of axillary lymph nodes dissection (ALND) is actively discussed. The condition of the axillary lymph nodes is considered one of the most important prognostic factors. Depending on this factor, the need for ALND is determined. ALND significantly increases the trauma of the surgery and leads to lymphedema, hematomas, limited movement in the shoulder joint, and other early and late postoperative complications, so the indications for it are limited. At this time, the accepted tactic is to perform a biopsy of the sentinel LN. In the absence of lesions of the axillary LN, ALND should be refused [55]. In addition, according to the study IBCSG 23-01, in patients with T1 with micrometastases in sentinel LN, the implementation of ALND did not affect the overall relapse-free survival at follow-up for 4 years [56]. An analysis of nine randomized clinical trials found that complete axillary lymph node dissection had no significant effect on overall survival, increased the risk of lymphedema, but reduced the risk of locoregional recurrence [57]. Another meta-analysis of several randomized trials found that ALND in patients with positive LN reduced the risk of local recurrence from 23% to 6% and 15-year mortality from 60% to 55% [58]. These results indicate the inexpediency of ALND surgery in some patients, even with lesions of sentinel LN. According to the recommendations of the American Society of Clinical Oncology, ALND should be performed only in those patients who have lesions of more than three sentinel LNs [59]. According to Yagata H. *et al.*, patients with TNBC are reasonable candidates for organ-preserving surgery due to the lack of widespread intraductal spread. The frequency of local recurrences after this treatment is not high, but the frequency of regional recurrences is increased, indicating the feasibility of biopsy of sentinel LN and ALND [60].

An equally important prognostic factor for local recurrence in breast cancer is the presence of cancer cells at the edges of the resection. The lowest risk of local recurrence is observed in the absence of cancer cells [61]. However, according to the results of the analysis of 589 women who underwent oncoplastic sparing surgery for breast cancer, it was found that the frequency of incomplete resection was 10.4% in total 18.9% in invasive carcinoma. Significant complications requiring hospitalization were reported in 9.2% of patients. The 5-year survival was 93.8%, recurrence-free – 91.7%. The frequency of local recurrences was 2.7%. It should be noted that the frequency of incomplete resection and severe complications were significantly lower after NACT [62], which indicates the impact on the treatment outcomes of other components of the treatment strategy.

Discussions on organ-preserving surgery in women with TNBC continue. According to many researchers, women with TNBC, even after radical surgery, have a fairly high frequency of locoregional and distant metastases and lower than other subtypes survival, leading to more active oncosurgical tactics and systemic treatment [7, 8]. Triple-negative status increases the risk of disease recurrence; however, some studies have shown that the incidence of locoregional recurrences and distant metastases was lower during resection than after mastectomy [46]. In addition, after organ-preserving surgery in women with TNBC or with other subtypes of cancer, no significant difference was found in terms of 5-year overall and recurrence-free survival. This, according to the authors, indicates the feasibility of organ-preserving surgery in women with TNBC [12].

There are no clear data on the impact on the treatment outcome of the type of surgery (RM or organ-sparing surgery) in patients with TNBC. The authors believe that the frequency of locoregional metastases is almost unaffected by the tumor subtype, but in TNBC more serious problem is isolated metastases, which indicates the need to improve systemic therapy [63].

#### Systemic therapy in TNBC treatment

Systemic therapy is an integral part of TNBC treatment. First, it is chemotherapy, which is carried out in neoadjuvant and adjuvant regimens and for palliative purposes [7]. Cyclophosphamide, Epirubicin, 5-fluorouracil were prescribed for TNBC, followed by the addition of Docetaxel for tumors larger than 2 cm and nodular cancer. NACT led to a positive response in 20% of patients who consider it a good prognostic sign. Good results were obtained with the use of platinum drugs. Systemic therapy was not used for tumors less than 1 cm, with no metastases observed for 4 years [60]. The feasibility of other chemotherapy drugs and targeted therapy is being discussed [64]. Lehmann *et al.* suggest that subtypes of TNBC should be considered when choosing a chemotherapeutic strategy. According to their data, Cisplatinum should be used for basal-like subtypes, NVP-BEZ235 (PI3K/mTOR inhibitor) and Dasatinib (abl/src inhibitor) for mesenchymal and mesenchymal stem subtypes; in the presence of androgen receptors – their antagonist bicalutamide [9].

Further study of the molecular genetic mechanisms of TNBC oncogenesis contributes to the development of new therapeutic strategies. In particular, the importance of microRNAs (miRNAs) and long noncoding RNAs (lncRNAs), which are involved in the regulation of gene expression, may be useful as biomarkers and possible targets of therapeutic action [65]. The prospects of immunotherapy in combination with lactic acidosis correction are discussed [66].

The frequency of complete pathomorphological response to NACT in TNBC was 23.0%, higher than in luminal subtypes of RM. The complete pathomorphological response to NACT was associated with decreased recurrence rates [19]. According to the results of a meta-analysis of 12 studies, which included more than 10 thousand patients with breast cancer, it was found that the frequency of complete pathomorphological response to treatment with Trastuzumab in neoadjuvant mode was the lowest in the luminal A subtype (7.5%), and in the most prognostically unfavorable subtypes was 50.3% in HER-2 positive patients and 33.6% in the triple-negative subtype [67]. In a study by Chen *et al.*, the frequency of complete pathomorphological response to NACT was 36% among 72 observations [68]. Therefore, it is considered appropriate to consider the response to NACT when developing a postoperative treatment strategy to address adjuvant CT and radiation therapy.

Brandão M. *et al.* considered it appropriate to use NACT in patients with aggressive breast cancer, particularly with luminal B, TNBC, and HER2+ subtypes. Surgical de-escalation is considered an argument in favor of NACT, which increases the possibilities for organ-preserving surgeries. In some patients, it reduces the need for complete axillary lymph dissection due to the conversion of N1 and N0. In addition, the response to NACT identifies patients with a high risk of relapse who need to plan additional therapeutic strategies, in particular, to determine the type of ACT. The authors believe that NACT should be the standard of therapy for TNBC and not an option to discuss the possibility of organ-preserving surgery. The lack of response to NACT allows the determination of optimal ACT, rather than prescribing chemotherapy blindly [69].

At the same time, according to Golshan M. *et al.*, NACT with Cisplatinum and Bevacizumab or Cisplatinum alone resulted in a significant increase in postoperative complications associated with wound healing. The use of Bevacizumab increased the number of complications after implantation and expanders. Among 28 patients who underwent NACT, organ-preserving surgery was performed in 13 (46%) and mastectomy in 15 (54%). Postoperative complications occurred in 11 (39%) patients [70]. According to a randomized study, NACT using Carboplatin in combination with Taxane and Trastuzumab in women with TNBC significantly increased the number of patients with a complete response but was associated with an increased incidence of systemic complications (neutropenia, anemia, and diarrhea) [71].

In addition to chemotherapy, adjuvant radiation therapy is considered useful in TNBC, which is used in 67% of patients with TNBC [7]. Some authors consider organ-preserving surgery followed by radiation therapy to be the “gold standard” for the treatment of early breast cancer. According to the results of several randomized studies, the long-term results of this method did not differ from the results of PME [72]. According to the results of several randomized studies, the long-term results of this method did not differ from the results of RM [72]. These and other researchers believe that oncoplastic surgery is less complex and traumatic but requires postoperative radiation therapy [36, 72]. On the other hand, according to Sinnott *et al.*, adjuvant radiation therapy promotes the development of capsular contracture in the prepectoral location of the implant (16.1% and 3.5%, respectively), and especially in the subpectoral location (52.2% and 2.9%, respectively). More often, capsular contracture occurred and was more severe with subpectoral implant placement [73].

According to the analysis of 20 randomized studies, Lee *et al.* found that the overall incidence of reconstructive insufficiency after endoprosthesis and radiation therapy was 17.6%, and capsular contracture – 37.5%. The development of postoperative complications depends on the duration of radiation therapy. The authors believe that radiation therapy during the first stage (during expander dermotension) increases the frequency of complications after surgery than when irradiated after implant placement but reduces the frequency of capsular contracture [74]. In another study, radiation therapy at the expander stage led to complications during a 6-year follow-up in 32% of patients, after implant placement – in 16.4% of patients. At the same time, in the group of patients with irradiation at the stage of expander, capsular contracture developed less often, and aesthetic results were better [75]. But in the meta-analysis of 20 randomized clinical trials, no patterns were found depending on the duration of radiation therapy. The authors concluded that it is necessary to consider the option of autoplasty instead of endoprosthesis when radiation therapy is needed [76].

Thus, in TNBC all basic treatment methods are used, including surgical, chemotherapy, and radiation therapy in complex or in combination. The treatment results depend on many factors that need to be considered when planning a treatment strategy in each case.

### Factors of negative prognosis of triple-negative breast cancer

The choice of a treatment strategy for oncopathology depends on individual characteristics of the patient, clinical and pathological features of the underlying disease and comorbidities, and the known effectiveness of treatment methods. The result of the influence of these features is the result of treatment, the analysis of which allows the identification of the most important prognostic factors.

#### Impact of treatment options on long-term result

In a study that analyzed the treatment results of 1,242 women with TNBC, 81% of patients received adjuvant therapy, no radiation therapy was prescribed. The average follow-up period was 78.3 months. The incidence of locoregional recurrence over 5 years was 4.2% and 5.4% after resection and after RM, respectively. The survival of women did not differ significantly depending on the extent of surgery but depended only on chemotherapy and the stage of the tumor [48]. According to Bayoumi *et al.*, 89% of patients received systemic therapy, and 63% of women received the PORT system (central catheter for chemotherapy). It was found that the frequency of local recurrence did not depend on the type of surgery but was lower among patients with PORT (7% vs. 19.5%) [18].

NACT in women with TNBC reduced the frequency of recurrent interventions [52]. The absence of pathomorphological response to NACT predicted the development of locoregional recurrence – with a complete response – 0%, in other cases – in 20% [68]. This indicator had the highest prognostic value after mastectomy [17]. James *et al.* consider radiation therapy, chemotherapy, and NACT to be significant prognostic factors [16]. A recent meta-analysis of 9 studies with a population of more than 4,000 breast cancer patients receiving NACT and organ-preserving surgical treatment identified 4 major risk factors for locoregional recurrence – estrogen-negative status, the presence of positive LN at diagnosis, residual positive LN after NACT, and more than 3 positive LN. Additional risk factors were: T3–T4 at diagnosis and residual breast tumor after NACT. It should be noted that this analysis did not take into account the molecular subtype of breast cancer [77].

In a study by Kümmel *et al.*, after surgical treatment and adjuvant therapy of early breast cancer, local recurrence was registered in 3.3%, locoregional – in 1%, and distant metastases – in 8% of cases. The 5-year survival rate for all was 92%. Additional risk factors were age less than 50 years, tumor size, luminal B subtype, and resection surgery. Factors of distant metastasis were lymphatic invasion and lack of systemic therapy [10]. In another study, CT did not affect survival over a 10-year follow-up [7].

Adjuvant radiation therapy is a common component of TNBC treatment, although its relevance after RM remains an unclear issue at this time. In particular, Haque *et al.* (2018) report that this type of treatment in patients with TNBC was useful only in T3; its effectiveness has not been proven in other stages [78]. Other researchers believe that radiation therapy in patients with TNBC is appropriate in the presence of two or more risk factors [53].

When studying the results of treatment of TNBC with radical mastectomy and NACT depending on the use of adjuvant radiation therapy, it was found that in the group with radiation therapy, the frequency of local recurrence during 5-year follow-up was 18.3%, without it – 52.2%; distant metastases – 45% and 69.1%, respectively. The authors believe that the rejection of adjuvant radiation therapy contributes to the development of locoregional recurrences and distant metastases. The greatest effect was found in patients with stage IIA [79]. According to a large-scale study (11,514 cases of TNBC), it was found that women who underwent organ-preserving surgery with radiation therapy had better survival rates than patients who underwent only radical mastectomy. This applies to the general cohort of patients and after stratification of patients by age, histology, stage of TNM, and tumor size, except for patients with TNBC stage I [80]. In patients with TNBC stage I–II after RM and adjuvant radiation therapy, the overall incidence of local recurrence was 2%, lymphatic metastasis – 3.4%, distant metastases – 9.0%, 7.4% of patients died. The five-year survival rate was 95.5% [81]. The absence of adjuvant radiation therapy after mastectomy was identified as an important prognostic factor [17]. This is consistent with another study in which ACT reduced the risk of recurrence, but the single prognostic negative recurrence factor was only RM without radiation therapy, which, according to the authors, indicates the feasibility of adjuvant radiation therapy after RM [49]. Vargo *et al.* reported the prognostic value of the absence of adjuvant radiation therapy after mastectomy [17], and Chen *et al.* found that adjuvant radiation therapy and the type of surgical treatment (mastectomy or organ-preserving surgery) did not significantly affect the development of relapses [68]. According to other data, independent risk factors for all forms of recurrence were N1, TNBC, and lack of radiation therapy [10].

#### Tumor and patients’ prognostic factors

In patients with early breast cancer (T1-2, N0-1) after NACT and mastectomy, independent risk factors for locoregional recurrence of the disease were stage N, lymphatic vascular invasion, and the degree of histological differentiation. Depending on these factors, the authors formed a group of low and high risk of recurrence. Adjuvant radiation therapy in the low-risk group did not affect the recurrence rate (3.3% with radiation therapy and 1.7% – without radiation therapy), in the high-risk group – reduced their frequency (21.8% and 42.2%, respectively) [82].

An additional factor in the negative prognosis of TNBC is overweight. Among 50 women included in the study, weight gain was found in 31 (62%). Progression during the follow-up period (31.1 months on average) was detected in 7 (14%) patients and was observed only in overweight women [83]. In addition, patients with TNBC worsen the prognosis with increasing frequency of distant metastasis androgen receptor expression (AP +) [84]. The presence of BRCA1 gene mutations, overexpression of human epidermal growth factor receptor 2, vascular endothelial growth factor-A, insulin-like growth factor-1 (IGF-1)/IGF-1 receptor and transforming growth factor-β1 are also associated with a worsening of TNBC prognosis [85].

Thus, the list of factors influencing the long-term results of treatment of breast cancer in general and its triple-negative subtype is quite broad, and they differ depending on the stage of the disease and the type of treatment strategy chosen, which must be considered when planning treatment.

## Conclusions

Triple-negative subtype is found in almost 20% of women with breast cancer. This subtype is characterized by an aggressive course with an increase in the frequency of locoregional and distant metastasis, leading to a dubious prognosis and, accordingly, encouraging an increase in the radicalism of surgical treatment. At the same time, this subtype is characterized by a high frequency of complete morphological response to NACT, which is a good prognostic sign. Therefore, in patients with TNBC, especially in the early stages, in the absence of locoregional and distant metastasis in addition to oncological radicalism, an important problem is to achieve good cosmetic results. The possibility and expediency of organ-preserving and reconstructive surgeries is still an unsolved problem. Along with the justification of the feasibility of organ-preserving surgeries, many oncosurgeons prefer RM with one- or two-stage reconstructive plastic surgery. Therefore, the choice of the optimal method of surgical treatment taking into account the individual characteristics of the patient and the tumor indications for chemotherapy and radiation therapy remains an unresolved issue and requires further research.

## Acknowledgments

### Conflict of interest

The authors declare that there is no conflict of interest.

### Authorship

AB, MK, VS, YuV contributed to conceptualization and methodology. AB, MK, VS, IZh, YuV contributed to investigation, data curation, formal analysis, and visualization. IZh, SK, VV, KO, OB, MS contributed to supervision and validation. All authors contributed to writing the original draft, reviewing, and editing the manuscript.
